# Impact of Treatment Duration in First-Line Atezolizumab Plus Chemotherapy in Extensive-Stage Small-Cell Lung Cancer: A Multicenter Real-World Retrospective Study

**DOI:** 10.3390/medicina61071230

**Published:** 2025-07-07

**Authors:** Mehmet Nuri Baser, Bilgin Demir, Gamze Serin Ozel, Gamze Gokoz Dogu, Serdar Karakaya, Mucahit Ugar, Naziye Ak, Ahmet Ozveren, Ufuk Camanlı, Olcun Umit Unal, Merve Turan, Esin Oktay

**Affiliations:** 1 Department of Medical Oncology, Faculty of Medicine, Adnan Menderes University, Aydin 09010, Turkey; bilgin287@hotmail.com (B.D.); drmerveturan@gmail.com (M.T.); esinct@gmail.com (E.O.); 2 Department of Medical Oncology, Faculty of Medicine, Pamukkale University, Denizli 20160, Turkey; gamze__239@hotmail.com (G.S.O.); ggokoz@pau.edu.tr (G.G.D.); 3 Department of Medical Oncology, Atatürk Sanatoryum Training and Research Hospital, Ankara 06290, Turkey; drserdarkarakaya@gmail.com; 4 Department of Medical Oncology, Institute of Oncology, Istanbul University, Istanbul 34093, Turkey; mucahitugar89@gmail.com (M.U.); naziye.ak@istanbul.edu.tr (N.A.); 5 Department of Medical Oncology, Acıbadem Kent Hospital, Izmir 35630, Turkey; ahmet.ozveren@kenthospital.com; 6 Department of Medical Oncology, Izmir City Hospital, Izmir 35540, Turkey; ufukcamanli@gmail.com (U.C.); drolcun@hotmail.com (O.U.U.)

**Keywords:** immunotherapy, chemotherapy, small-cell lung cancer

## Abstract

*Background and Objectives:* Small-cell lung cancer (SCLC) is an exceedingly aggressive neoplasm distinguished by an unfavorable prognosis. Recent studies have confirmed chemo-immunotherapy as the conventional first treatment for extensive-stage small-cell lung cancer (ES-SCLC), but the impact of treatment duration remains unclear. The goal of this study was to find out how the length of treatment affected progression-free survival (PFS) and overall survival (OS) in patients with ES-SCLC who were receiving first-line atezolizumab plus chemotherapy. *Materials and Methods:* This retrospective multicenter study comprised 82 patients from six oncology centers in Turkey between 2017 and 2024. Patients were categorized into two categories according to the quantity of chemotherapy cycles they had undergone: standard treatment (≤4 cycles) and extended treatment (≥5 cycles). For the purpose of analyzing survival outcomes and related clinical determinants, as well as the demographic structures and features of the patients, both univariate and multivariate Cox regression models were utilized. *Results:* The median number of atezolizumab cycles was 8 (1–63). OS was 29.46 months after 15.8 months of follow-up, while PFS was 10.63 months. When comparing the two groups, we found no statistically significant differences in either PFS (*p* = 0.952) or OS (*p* = 0.374). Significant associations with OS were seen in the standard therapy group for both ECOG PS 1 (*p* = 0.028). Thoracic radiation considerably decreased progression risk (HR = 0.41, *p* = 0.031) in the extended group. *Conclusions:* While prolonging chemo-immunotherapy beyond four cycles did not significantly improve survival, the selected patient subgroups may benefit from personalized approaches. Thoracic radiotherapy emerged as a key modifier of outcome.

## 1. Introduction

Lung cancer ranks second in male and female incidence rates in the United States [1] and is responsible for around 20.4% of all cancer-related fatalities in 2024 [2]. About 13–15% of lung cancers are small-cell lung cancers (SCLCs), which are aggressive neuroendocrine tumors [3]. Approximately 70% of individuals identified with SCLC have advanced to an advanced stage, meaning they have a poor prognosis with a median survival of less than one year [4].

Four to six cycles of etoposide cisplatin (EC) or etoposide carboplatin (EP) were administered as the first therapy for extensive stage small-cell lung cancer (ES-SCLC) [5]. Currently, the conventional first-line therapy for ES-SCLC is platinum and etoposide chemotherapy, with a preference for carboplatin over cisplatin because of its reduced adverse effects in clinical practice as an institutional policy [6], as well as programmed death ligand 1 (PD-L1)-blocking antibodies, atezolizumab, or durvalumab in combination with chemotherapy, according to the IMpower 133 and CASPIAN clinical trials, respectively [7]. In the Impower 133 trial, the addition of atezolizumab to 4 cycles of platinum-doublet chemotherapy followed by maintenance atezolizumab treatment improved overall survival (OS) from 10.3 to 12.3 months [8]. However, this effect of immunotherapy was limited to only 2 months, and platinum-based chemotherapy remains an indispensable component of treatment in these patients.

The optimal duration of this combination therapy remains undefined in real-world clinical practice. The purpose of this research is to investigate whether or not prolonging chemotherapy beyond the conventional four cycles has an impact on survival rates in patients with SCLC who are being treated with atezolizumab.

## 2. Materials and Methods

### 2.1. Patients

This research was authorized by Adnan Menderes University Ethical Review Board in accordance with the Declaration of Helsinki (Date: 9 January 2025; Decision no: 14-2023/93).

This research is a multicenter retrospective analysis including a total of 6 cancer diagnosis and treatment sites covering different provinces in Turkey. Between 1 January 2017 and 1 June 2024, screenings were conducted on patients who had been diagnosed with an extensive stage of SCLC according to the staging criteria developed by the Veterans Administration Lung Cancer Study Group (VALSG). The diagnosis was validated by the use of histology or cytology. After screening, comprehensive examination and imaging were employed to rigorously enroll individuals who were at least 18 years old, totaling 82 patients, and receiving atezolizumab in combination with EC or EP in first-line therapy. Patients with incomplete response assessment, missing laboratory parameters, and patients without pretreatment positron emission tomography-computed tomography (PET/CT) imaging were excluded. Prior to treatment initiation, all patients underwent physical examination, systemic examination using PET/CT, and cerebral magnetic resonance imaging (MRI). PET/CT and, when necessary, thoracic CT were used for response assessment. Regular follow-up for disease progression was performed every 3 months until the end of follow-up or patient death. In order to evaluate the effectiveness of the treatment intervention, the Response Evaluation Criteria in Solid Tumors (RECIST version 1.1) were applied. Illnesses that were progressing (PD), disease that was stable (SD), partial response (PR), or complete response (CR) were the categories that were presented for analysis. Throughout the course of treatment and for a period of thirty days after treatment, adverse events that were associated with the treatment were recorded and evaluated in accordance with the National Cancer Institute Common Terminology Criteria for Adverse Events version 5.0.

Information on the patients’ demographics, clinicopathologic conditions, response rates, and survival rates was documented in a retrospective manner using a computer system.

### 2.2. Treatments

The PD-L1 inhibitor atezolizumab was administered to patients in conjunction with EC or EP on a 21-day cycle in this study. Every 21 days, patients in each cycle received doses of cisplatin or carboplatin intravenously (75 mg/m^2^ or 5 mg/mL per minute, respectively), etoposide intravenously (100 mg/m^2^ on days 1–3), and atezolizumab intravenously (1200 mg on day 1). In this multicenter retrospective analysis, the selection of the platinum agent was based on the clinical assessment of the treating physicians and the preferences of the institutions involved. Once the induction phase was complete, patients continued to receive 1200 mg of atezolizumab intravenously every three weeks during the maintenance phase. This dosage continued until the illness progressed, the patient had severe side events according to RECIST criteria, or they requested to stop therapy.

Accordingly, those who received 4 cycles or fewer of treatment were defined as the standard treatment group, and those who received 5 cycles or more were defined as the extended treatment group.

### 2.3. Statistical Analysis

We used “IBM SPSS Statistics for Windows, Version 25.0 (Statistical Package for the Social Sciences, IBM Corp., Armonk, NY, USA)” to run our statistical analyses. For categories, descriptive statistics are given as n and %, whereas for continuous variables, they are given as Mean ± SD and median (min–max). Survival and progression-free survival times were compared among clinical groups using the Kaplan–Meier approach. Presented here are the results of multivariate Cox regression analyses for a wide variety of clinical variables that have been shown to influence mortality risk and illness progression. Statistical significance was determined by a *p*-value lower than 0.05.

## 3. Results

### 3.1. Patients’ Characteristics and Efficacy

The median age of the patients participating in the research was 64 years, with a range of 45 to 82 years. A total of 58.5% of the patients were aged 65 years or younger, while 41.5% were over 65 years of age. The gender distribution was 20.7% female and 79.3% male. The presence of concomitant comorbidities was detected in 68.2%. Chronic obstructive pulmonary disease (COPD), diabetes mellitus (DM), hypertension (HT), and coronary artery disease (CAD) were seen respectively in 25.6%, 17.1%, 7.3%, and 18.2%. Smoking was present in 87.8% of patients and absent in 12.2%. Histopathologic examination revealed SCLC in 98.8% of patients and large-cell neuroendocrine carcinoma (LCNEC) in 1.2%. In terms of disease status, 93.9% had de novo disease, while 6.1% had recurrent extensive-stage disease (Table 1). As seen in Table 2, 84% of patients received the EP regimen with immunotherapy (IO), while 16% were treated with the EC regimen. In terms of the total number of chemotherapy (CT) and IO cycles, 45.1% of the patients received normal treatment and 54.9% received prolonged treatment. The median number of atezolizumab cycles administered was 8 (1.0–63.0). Regarding radiotherapy (RT) applications, 28.4% of patients received brain RT, 28.4% received thoracic RT, and the remaining patients did not receive these treatments. (Table 1). In terms of the best response to first-line treatment, the objective response rate (ORR) was 70.8%, whereas advancement occurred in 18.3% of cases.

### 3.2. Survival Outcomes

**Figure 1:**
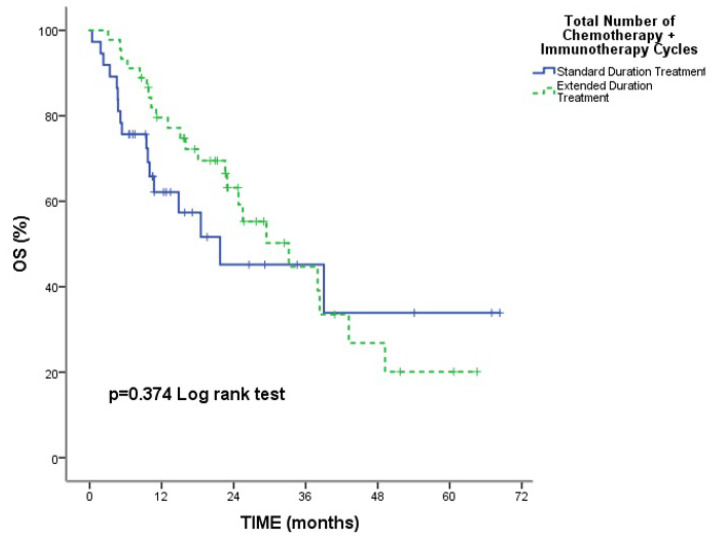
Comparison of Median OS Between Standard and Extended Treatment Groups.

**Figure 2:**
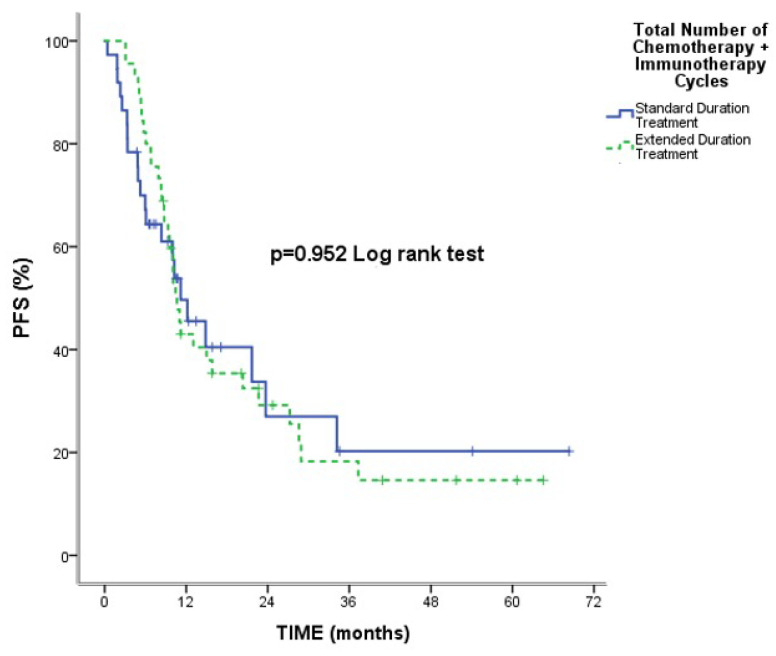
Comparison of Median PFS Between Standard and Extended Treatment Groups.

The median follow-up time was 15.81 months, with a range of 0.40 to 68.33 months, and the death rate was 48.8%. Overall median PFS and OS were 10.63 months (95% CI: 8.62–12.64) and 29.46 months (95% CI: 17.35–41.58). No statistically significant change was seen in median OS [21.73 months (95% CI: 0.55–42.90) and 33.20 months (95% CI: 19.48–46.91), respectively] and median PFS [11.16 months (95% CI: 6.16–16.17) and 10.63 months (95% CI: 9.21–12.05), respectively] between the standard and extended treatment groups (*p* = 0.374; *p* = 0.952, respectively) (Figure 1 and Figure 2).

Univariate analysis revealed that median OS (months) was statistically significant for ECOG-PS 1 (Eastern Cooperative Oncology Group Performance Score) (*p* = 0.028) in the standard therapy group. Age, gender, smoking, total number of metastatic sites at the time of diagnosis, CT regimen received with IO, and history of RT to the brain and thorax did not differ in both groups.

Consequent to univariate analysis, the median PFS in the extended treatment group was statistically significant for the total number of metastatic sites at diagnosis (*p* = 0.010) and thoracic radiation (*p* = 0.032). Age, gender, ECOG, smoking, CT regimen received with IO, and history of brain RT did not differ in both groups. The results of the multivariate Cox regression analysis for factors associated with mortality in the standard treatment group are summarized in Table 3. The multivariate Cox regression model findings indicate the progression risk associated with several clinical factors in the extended treatment group: having 1 total metastatic area at diagnosis increased the risk of progression 5.24-fold (HR: 5.24, 95% CI: 1.71–16.09, *p* = 0.004), having 2 total metastatic areas at diagnosis was 3.51-fold (HR: 3.51, 95% CI: 1.03–12.01, *p* = 0.045), and having 4 total metastatic sites at diagnosis was 13.37-fold (HR: 13.37, 95% CI: 3.45–51.69, *p* < 0.001). However, it was found to decrease the risk of progression in those who received thoracic RT (HR: 0.41, 95% CI: 0.17–0.92, *p* = 0.031) (Table 4).

### 3.3. Toxicities

Table 5 displays treatment-related adverse events. Most hematologic adverse events were Grade 1 or 2. Among these, anemia (51.2%), neutropenia (39.0%), and fatigue (59.8%) were the most common Grade 1 or 2 events. Regarding severe (Grade 3 or 4) hematologic toxicities, neutropenia (22.0%), anemia (13.4%), and fatigue (15.9%) were the most frequent. Additionally, 72.7% of pneumonia cases were Grade 1 or 2, while 27.3% were Grade 3 or 4.

## 4. Discussion

We analyzed real-life data extensively to determine the safety and effectiveness of CT with atezolizumab treatment length as a first-line therapy for ES-SCLC. Median PFS and OS rates were not significantly different between patients receiving conventional therapy and those receiving prolonged treatment (*p* = 0.952 and *p* = 0.374, respectively). The extent of metastatic sites was linked to progression risk in patients receiving extended treatment. A history of thoracic RT decreased the risk of progression in those who received extended treatment. Hematologic side effects were the most common adverse events, with neutropenia being the leading severe toxicity, while febrile neutropenia was less frequent.

In the IMpower 133 study, where standard 4 cycles of chemotherapy and atezolizumab were used, there was a median of 12.3 months of OS and 5.2 months of PFS [8]. In our study, both the overall and the standard and extended treatment groups had longer median PFS and OS. In another study, there was a median of 5.2 months of PFS and a median of 11.3 months of OS [9], while in Lee et al.’s study, the median PFS and OS were similar (4.6 months and 12 months, respectively) [10], which are shorter compared to our study. The longer PFS observed in our study compared to the IMpower 133 and Şahin et al.’s study (with ORRs of 60.2% and 63.6%, respectively) may be due to the higher ORR of 70.8% in our study. The longer OS in our study may be due to the small number of patients, the relatively younger patients, and the presence of patients who received thoracic RT. In Bilgin et al.’s study, ORR was 87.5% and median PFS was 9.5 months, while OS was 30.1 months [11]. Its OS was longer, and its ORR was higher than ours.

The median number of atezolizumab cycles in the IMpower 133 study was 7 [8], whereas in our study it was 8. In the IMpower 133 study, there was a contribution to OS in patients over 65 years old and those with ECOG-PS 1. In our study, while there was an OS benefit in patients with ECOG-PS 1 receiving standard treatment, this benefit was not observed in those receiving extended treatment. Choi et al. found that an ECOG PS of 2–3 correlated with worse prognosis for both PFS and OS [12]. In our study, no relationship with age was observed during either treatment period. According to the results of a meta-analysis conducted by Yang et al., the relationship between gender and age with the response to immunotherapy was not demonstrated in our study [13].

Gürbüz et al. found that lower PFS and OS are associated with more metastatic locations [14]. Additionally, in the Phase 3 first-line therapy trial with patients who had ES-SCLC and were given pembrolizumab with CT, the median PFS in the pembrolizumab arm was longer, independent of the number of metastatic sites; however, OS was superior in those with three or more metastatic locations [15]. Our findings established that among patients receiving extended treatment, the number of metastatic sites increased the risk of progression, but there was no relationship with OS. The current situation may be related to the increase in disease burden in extended treatment areas. The quantitative rise in metastatic sites has not correlated with a corresponding escalation in progression risk; this phenomenon may stem from variations in patient distribution. A meta-analysis revealed that smoking is an independent adverse risk factor in individuals with SCLC [16], and Gürbüz et al.’s study supported this [14]. Nonetheless, our investigation did not identify such a link, and the proportion of non-smoking patients was more than that reported by Gürbüz et al. (12.2% versus 2.8%, respectively) [14].

Several factors may explain why the benefit of thoracic RT was observed only in the extended treatment group. Evidence suggests that consolidation thoracic RT can be useful in patients with ES-SCLC, and in the study by Bonanno et al., it was suggested that chemoimmunotherapy could induce RT responses [17,18]. In our study, similarly, the use of prolonged chemoimmunotherapy may have resulted in a reduction in the risk of progression due to its ability to increase response rates, and this effect may not have occurred in patients receiving short-term treatment. In addition, in the group of patients who continued treatment above standard therapy, better disease control may have contributed to the reduced risk of progression in this group.

A study assessing the incorporation of durvalumab, an immunotherapy, into the platinum/etoposide regimen for extensive-stage SCLC revealed no disparity in OS across the groups treated with carboplatin and cisplatin [19]. In the IMpower 133 study, only the use of carboplatin was permitted [8]. In our study, atezolizumab was combined with both carboplatin and cisplatin, and CT was administered for more than 4 cycles in some patients. However, the difference in platinum regimens (carboplatin vs. cisplatin) did not result in any difference in overall survival in either the standard treatment group or the extended treatment group. In our study, there were no side effects that caused treatment discontinuation or death, and the side effects of all grades were manageable.

## 5. Conclusions

In conclusion, our study indicates that therapeutic success in the extended first-line therapy of ES-SCLC may be affected by patient selection criteria, including ECOG-PS, metastatic status, and prior thoracic RT history. Therefore, it highlights the results for the use of treatments in a broader therapeutic context and emphasizes the importance of personalized treatment approaches for this patient group. Determining the ideal treatment duration and evaluating the effectiveness of the therapeutic environment require additional prospective studies with larger patient groups and real-world data.

## Figures and Tables

**Table 1:** Characteristics of the patients.

Parameters	N	%
Age		
Median (min–max)	64.0 (45–82)	
≤65	48	58.5
>65	34	41.5
Gender		
Female	17	20.7
Male	65	79.3
Comorbidity		
No	26	31.8
Yes	56	68.2
ECOG-PS		
0	31	37.8
1	43	52.4
2	7	8.5
3	1	1.2
Smoking		
No	10	12.2
Yes	72	87.8
Histopathology		
SCLC	81	98.8
LCNEC	1	1.2
Disease condition		
De novo	77	93.9
Recurrent extensive stage	5	6.1
Total number of metastatic sites at the time of diagnosis		
1	16	19.5
2	28	34.1
3	20	24.4
4 and more	18	22.0
Surrenal metastasis		
No	58	70.7
Yes	24	29.3
Brain metastasis		
No	65	79.3
Yes	17	20.7
Liver metastasis		
No	58	70.7
Yes	24	29.3
Peritoneal metastasis		
No	80	97.6
Yes	2	2.4
Lung metastasis		
No	32	39.0
Yes	50	61.0
Bone metastasis		
No	36	43.9
Yes	46	56.1
Non-regional lymph node metastasis		
No	30	36.6
Yes	52	63.4
Duration of follow-up (months)		
Median (min–max)	15.81 (0.40–68.33)	
Brain RT		
No	59	72.0
Yes	23	28.4
Thoracic RT		
No	58	71.6
Yes	23	28.4

ECOG-PS: Eastern Cooperative Oncology Group Performance Score, SCLC: Small-cell lung cancer, LCNEC: Large-cell neuroendocrine carcinoma, RT: Radiotherapy.

**Table 2:** Characteristics of therapy and objective tumor responses.

Parameters	N	%
Chemotherapy Regimen with IO		
EP	68	84.0
EC	13	16.0
Total Number of Chemotherapy + IO Cycles		
Standard Treatment	37	45.1
Extended Treatment	45	54.9
Total Number of Chemotherapy + IO Cycles		
Median (min–max)	6.0 (0–9.0)	
Total Number of Atezolizumab Cycles		
Median (min–max)	8.0 (1.0–63.0)	
Best Response to First-Line Therapy		
CR	13	15.9
PR	45	54.9
SD	9	11.0
Progression	15	18.3

IO: Immunotherapy EC: Etoposide cisplatin EP: Etoposide carboplatin CR: Complete Response, PR: Partial Response, SD: Stable Disease.

**Table 3:** Multivariate Cox Regression Analysis of Clinical Variables Associated with Mortality in the Standard Treatment Group.

	Standard Treatment	
Parameters	HR (%95 CI)	*p*
Age		
≤65	ref	0.666
>65	1.30 (0.38–4.38)	
Gender		
Female	ref	0.054
Male	0.23 (0.05–1.02)	
ECOG-PS		0.153
0	ref	
1	4.58 (0.47–44.51)	0.189
2	12.32 (0.91–166.85)	0.059
Smoking		
No	ref	0.107
Yes	0.06 (0.01–1.83)	
Chemotherapy Regimen with IO		
EP	ref	0.150
EC	0.17 (0.02–1.89)	
Thoracic RT		
No	ref	0.320
Yes	0.19 (0.01–5.01)	

−2 Log Likelihood = 77.44, *p* = 0.031 ECOG-PS: Eastern Cooperative Oncology Group Performance Score, IO: Immunotherapy, EC: Etoposide cisplatin, EP: Etoposide carboplatin, RT: Radiotherapy.

**Table 4:** Multivariate Cox Regression Analysis of Clinical Variables Associated with Progression Risk in the Extended Treatment Group.

	Extended Treatment	
Parameters	HR (%95 CI)	*p*
Smoking		
No	ref	0.302
Yes	0.59 (0.21–1.61)	
Total number of metastatic sites at the time of diagnosis		0.002
1	ref	
2	5.24 (1.71–16.09)	0.004
3	3.51 (1.03–12.01)	0.045
4	13.37 (3.45–51.69)	<0.001
Thoracic RT		
No	ref	0.031
Yes	0.41 (0.17–0.92)	

−2 Log Likelihood = 174.27, *p* < 0.001 RT: Radiotherapy.

**Table 5:** Treatment-Related Adverse Events.

Event	Grade 1 or 2 N (%)	Grade 3 or 4 N (%)
Anemia	42 (51.2)	11 (13.4)
Neutropenia	32 (39.0)	18 (22.0)
Thrombocytopenia	17 (20.7)	8 (9.8)
Febrile Neutropenia	6 (7.3)	4 (4.9)
Fatigue	49 (59.8)	13 (15.9)
Nausea	34 (41.5)	7 (8.5)
Vomiting	19 (23.2)	2 (2.4)
Acute Kidney Injury	9 (11.0)	1 (1.2)
Elevated Hepatic Transaminases	10 (12.2)	1 (1.2)
Diarrhea	15 (18.3)	2 (2.4)
Hypothyroidism	16 (19.5)	1 (1.2)
Pneumonia	8 (9.8)	3 (3.7)

## Data Availability

The data sets and analyses from the study can be obtained from the corresponding author upon reasonable request.
